# Integrating fecal metabolomics and intestinal microbiota to study the mechanism of cannabidiol in the treatment of idiopathic pulmonary fibrosis

**DOI:** 10.3389/fphar.2024.1358626

**Published:** 2024-02-06

**Authors:** Mengdi Sun, Feiyu Zhang, Fang Lu, Donghua Yu, Yu Wang, Pingping Chen, Shumin Liu

**Affiliations:** Institute of Traditional Chinese Medicine, Heilongjiang University of Chinese Medicine, Harbin, Heilongjiang, China

**Keywords:** cannabidiol, pulmonary fibrosis, metabolomics, intestinal microbiota, mechanism

## Abstract

**Introduction:** Idiopathic pulmonary fibrosis is a chronic interstitial lung disease characterized by excessive deposition of extracellular matrix. Cannabidiol, a natural component extracted from plant cannabis, has been shown to have therapeutic effects on lung diseases, but its exact mechanism of action is unknown, hindering its therapeutic effectiveness.

**Methods:** To establish a pulmonary fibrosis model, combined with UPLC-Q-TOF/MS metabolomics and 16S rDNA sequencing, to explore cannabidiol’s mechanism in treating pulmonary fibrosis. The rats were randomly divided into the control group, pulmonary fibrosis model group, prednisone treatment group, and cannabidiol low, medium, and high dose groups. The expression levels of HYP, SOD, and MDA in lung tissue and the expression levels of TNF-α, IL-1β, and IL-6 in serum were detected. Intestinal microbiota was detected using UPLC-QTOF/MS analysis of metabolomic properties and 16S rDNA sequencing.

**Results:** Pathological studies and biochemical indexes showed that cannabidiol treatment could significantly alleviate IPF symptoms, significantly reduce the levels of TNF-α, IL-1β, IL-6, MDA, and HYP, and increase the expression level of SOD (*p* < 0.05). CBD-H can regulate *Lachnospiraceae_NK4A136_group*, *Pseudomonas*, *Clostridia_UCG-014*, *Collinsella*, *Prevotella*, *[Eubacterium]_coprostanoligenes_group*, *Fusobacterium*, *Ruminococcus*, and *Streptococcus*, it can restore intestinal microbiota function and reverse fecal metabolism trend. It also plays the role of fibrosis through the metabolism of linoleic acid, glycerol, linolenic acid, and sphingolipid.

**Discussion:** Cannabidiol reverses intestinal microbiota imbalance and attenuates pulmonary fibrosis in rats through anti-inflammatory, antioxidant, and anti-fibrotic effects. This study lays the foundation for future research on the pathological mechanisms of IPF and the development of new drug candidates.

## 1 Introduction

Idiopathic pulmonary fibrosis (IPF) is a chronic and progressive pulmonary interstitial disease with unknown causes, mainly occurring in middle-aged and elderly people. The prognosis is poor, and the median survival is only 3–5 years. The main feature of IPF is tissue scarring caused by excessive deposition and excessive repair of extracellular matrix (ECM) (Mei et al., 2022). It is currently recognized that pulmonary fibrosis is an epithelial-driven disease, the main mechanism of which is epithelial-mesenchymal transition (EMT). Sustained epithelial cell injury would cause abnormal activation of epithelial cells and then secrete a large amount of transforming growth factor-β (TGF-β) (Inui et al., 2021), which was characterized by interstitial scar formation and irreversible decline in lung function (Strykowski and Adegunsoye, 2023). Smoking, air pollution, and occupational exposure are considered to be risk factors for IPF (Schäfer et al., 2020). Data show that pulmonary fibrosis affects about 3 million people in the world, and with aging and serious air pollution, global morbidity and mortality are increasing year by year (Moss et al., 2022). At present, Nidanib and pirfenidone have been approved by the FDA and are clinically used to treat pulmonary fibrosis, but they can only delay the fibrosis process, and the prognosis is still poor, and long-term use will cause gastrointestinal and other adverse reactions (Spagnolo et al., 2018). Therefore, it is necessary to further study the physiological and pathological mechanisms of pulmonary fibrosis and actively seek new treatment strategies.

Cannabidiol (CBD) is a non-psychoactive derivative of the cannabis plant (Meissner and Cascella, 2023) and has been valued for its anti-anxiety, anti-emetic, anti-inflammatory, and anticancer properties (Legare et al., 2022). As a powerful antioxidant, CBD acts on a variety of receptor sites, directly or indirectly causing a wide range of anti-inflammatory and immunomodulatory effects (Atalay et al., 2019). In 2018, the FDA approved the use of CBD to treat epilepsy in children, especially Dravet syndrome (Vossler et al., 2018; Reddy, 2023). Previous research has shown that CBD can reduce the production of related cytokines in animal models of chronic asthma. Recently, it has been established that CBD is involved in regulating inflammatory responses, including inflammatory lung diseases, and has a positive effect on acute and chronic inflammation (Eskander et al., 2020; Urits et al., 2020; Porter et al., 2021). Previous studies of our research group found that CBD can reduce the expression of TGF-β and α-SMA in rat lung tissue; the content of TGF-β1, α-SMA, and NF-κB p65m RNA in lung tissue was decreased, and the expression level of Nrf2 m RNA was increased, especially in the high-dose group (Sun et al., 2023). In addition, CBD is well tolerated with no significant side effects, and it may also be an effective candidate for the treatment of pulmonary fibrosis. However, there is little research on the pharmacological activity and mechanism of CBD in the treatment of IPF.

There is evidence that IPF is inextricably linked to the gut microbiome, which is considered an indispensable “metabolic organ” that plays a crucial role in maintaining human health or causing disease (Chen et al., 2021). Significant changes in gut microbiota composition have been observed in IPF patients, and restoring gut microbiota imbalance through dietary probiotic inulin supplementation with alpha or linolenic acid-rich flaxseed oil can help improve IPF (Abidi et al., 2020). Previous studies have suggested that immune cells and cytokines, which are prompted by the gut microbiota and its metabolites, including SCFAs, may penetrate the systemic circulation via the blood and lymphatic system. This process plays a crucial role in controlling immune and inflammatory reactions within the lungs, thereby impacting respiratory wellbeing and ailments (Ashique et al., 2022). On the other hand, the imbalance of intestinal microbiota can stimulate inflammatory activity and disrupt energy balance, affecting respiratory immunity and barrier function (Chunxi et al., 2020). Likewise, specific plant life can aid in the body’s maintenance of a balanced immune system by diminishing the inflammatory response and assisting the lungs in combatting infection (Ma et al., 2022).

Sequencing of the 16S rRNA gene enables precise identification of the structure of gut microorganisms. Metabolomics offers a comprehensive assessment of changes in metabolites during the occurrence and progression of diseases. The utilization of 16S rRNA gene sequencing and metabolomics has become widespread in exploring the mechanisms of diseases and drug therapies, unraveling the pathogenic processes, and identifying disease biomarkers.

**FIGURE 1:**
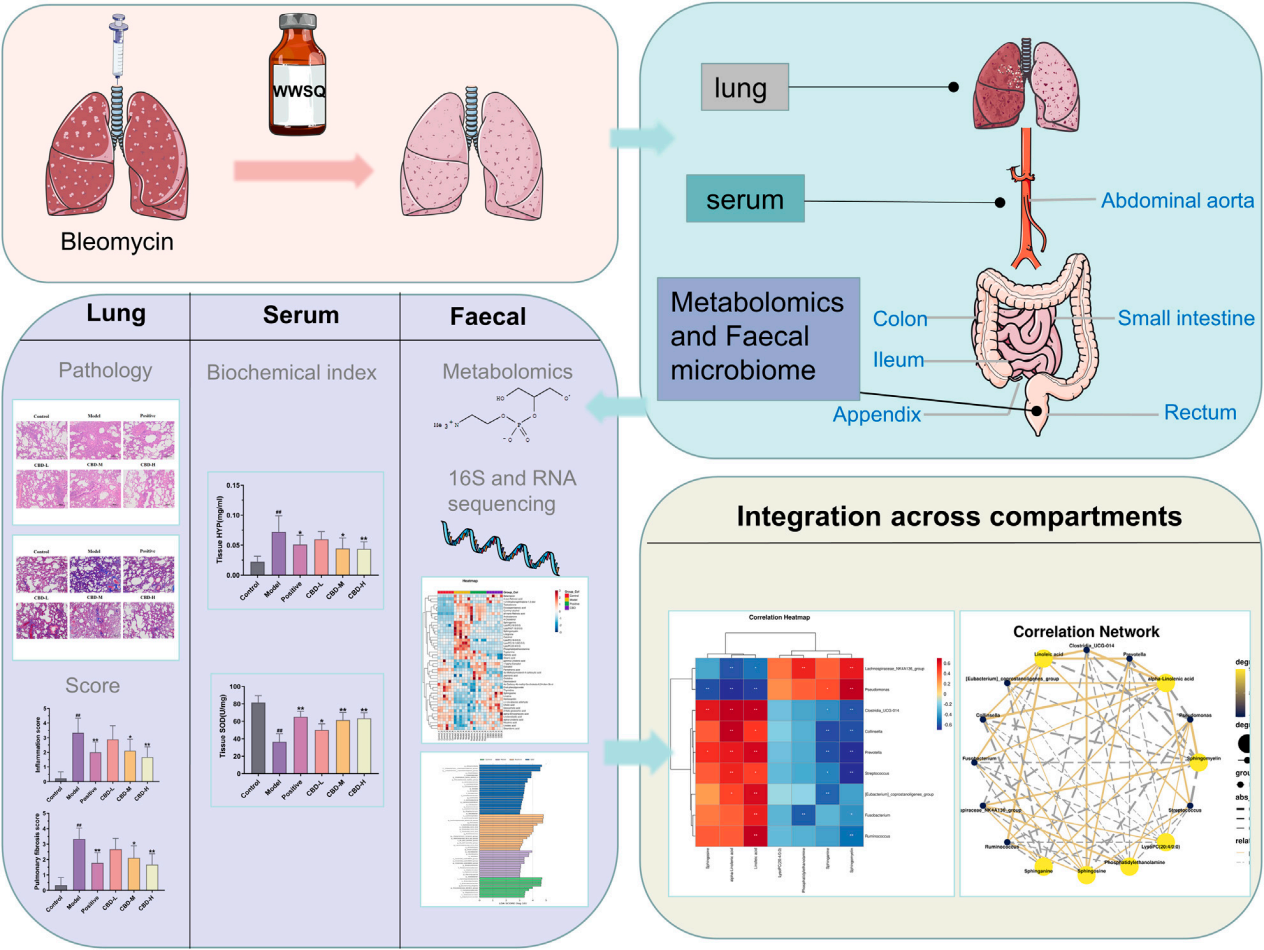
Research design. After 28 days of administration, samples were obtained from lung tissue and arterial serum stool. See Method for experimental procedure and analysis.

Consequently, in conjunction with fecal metabolomics and gut microbiota analysis, this current investigation more comprehensively illustrates CBD’s protective mechanisms in IPF rat models (Figure 1). The findings of this study provide experimental evidence and scientific support for the lung-protective effects of CBD, as well as offer fresh perspectives for the rational development and utilization of CBD resources in the pharmaceutical and food sectors.

## 2 Materials and methods

### 2.1 Experimental materials

CBD was purchased from Xi’a Lvruquan Biotechnology Co., LTD. (Lot number: LRQ221103-1); ELISA kits for TNF-α (ml002859), IL-1β (ml037361) and IL-6 (ml064292) were purchased from Shanghai Enzyme-Linked Biotechnology Co., LTD. Bleomycin (lot number: Z8020) was purchased from Solebol Biotechnology Co., LTD. Prednisone acetate tablets (lot number: LA22255) were purchased from Zhejiang Sienju Pharmaceutical Co., LTD. Acetonitrile (chromatographic grade) (batch number: A996-4), methanol (chromatographic grade) (batch number: 67-52-1, Dikma Technology Company); Ultra high liquid chromatography-time-of-flight Tandem Mass Spectrometer (Waters, United States); KDC-160HR high-speed refrigerated centrifuge (China University of Science and Technology Innovation Co., LTD.).

Preparation of cannabidiol (CBD): Preparation of 0.5% sodium carboxymethyl cellulose (CMC-Na) three-steam aqueous solution, take 108 mg cannabidiol dissolved in 10 mL of the above solution to make a suspension, the concentration is 10.8 mg/mL, diluted during use.

### 2.2 Experimental animals

Sixty SPF grade SD male rats, body weight (190 ± 20) g, were purchased from the Experimental Animal Center of Heilongjiang University of Traditional Chinese Medicine, No. 2022062023, and were allowed to enter the experimental stage after 7 days of adaptive feeding. All relevant operations of this experiment were conducted according to SPF laboratory regulations and requirements and were approved by the Experimental Animal Ethics Committee of Heilongjiang University of Chinese Medicine.

A total of sixty rats were randomly divided into four groups: normal control group, model group, prednisone group (dosed at 3.15 mg/kg), and CBD groups with low, medium, and high doses (12, 36, 108 mg/kg) (Ye et al., 2019). Weekly monitoring was conducted for the rats’ body weight and diet. Pulmonary fibrosis was induced in the rats by administering bleomycin through intratracheal injection. The rats were injected intraperitoneally with 3% sodium pentobarbital (40 mg/kg), the neck skin was cut open, the trachea was exposed, a 1 mL syringe was used to gently insert the trachea of the rats, and cotton wool was placed at the outer orifice of the syringe tube to check whether the insertion was successful. Bleomycin (5 mg/kg) was quickly injected and 0.2 mL of air was continued to be injected to ensure that the liquid reached the lungs evenly. The rat skin was sutured. Starting from the second day after modeling, the drug was given once a day by gavage for 28 days. Lung tissues were collected for biochemical and histopathological analysis. Blood was collected from the abdominal aorta and serum was extracted. The cecum of the rat was removed immediately and the contents were collected and stored in a −80°C refrigerator for intestinal microbiota and metabolomics studies.

### 2.3 Determination of pulmonary organ coefficient and wet/dry mass (W/D) ratio of lung tissue

The rats were dissected, the complete lung tissue was removed, the surface blood was drained, the lung mass was weighed, and the lung coefficient of the rats in each group was calculated: organ coefficient = organ mass/body mass ×100%. In addition, the wet mass of the upper lobe of the right lung of rats was weighed, and baked in a 60°C oven for 72 h, and its dry mass was weighed, and the moisture content of the lung was evaluated by calculating the wet/dry weight ratio.

### 2.4 Histological examination

Paraffin sections were stained with hematoxylin-eosin and Masson according to standard protocols. Lung injury index or fibrosis was assessed according to the Ashcroft histopathological scale (Ashcroft et al., 1988).

### 2.5 Biochemical analysis

The contents of TNF-α, IL-1β, and IL-6 in serum and SOD, MDA, and HYP in lung tissue of rats were detected by enzyme-linked immunosorbent assay (ELISA).

### 2.6 Metabolomics analysis

#### 2.6.1 UPLC-Q-TOF/MS

The UPLC conditions employed in this study involved the utilization of state-of-the-art ultra-high-performance liquid chromatography combined with a tandem time-of-flight mass spectrometry system (UPLC-Q-TOF/MS). The chromatographic separation was executed using a C18 column with dimensions of 2.1 mm * 100 mm and a particle size of 1.7 μm. The C18 column used in this analysis was manufactured by Waters Corporation, a renowned company based in Milford, United States. The mobile phase composition consisted of acetonitrile (containing 0.05% formic acid) as mobile phase A, while mobile phase B comprised water (containing 0.05% formic acid). A gradient elution approach was implemented, where the following elution profile was employed: 0–8 min: 98%–60% mobile phase B; 8–10 min: 60%–2% mobile phase B; 10–13 min: 2%–0% mobile phase B; 13–14 min: 0%–98% mobile phase B; 14–17 min: 98%–98% mobile phase B. The flow rate utilized during the analysis was 0.40 mL/min. The injection volume for each sample was set at 2 μL. The column temperature was maintained at 40°C, whereas the sample chamber temperature was kept at 5°C.

Mass spectrum conditions: diode array detector full wavelength scanning, UV detector effluent directly into the mass spectrum system without shunt detection. The detection was performed using both positive and negative ion modes in the Electrospray ion source (ESI). The concentration of the locking mass was 2.0 μg·L-1, while the flow rate was set at 40 μL·min-1. The desolvent temperature was maintained at 350°C, and the desolvent gas flow was set to 750.0 L/h. The ion source temperature was set at 110°C, with a cone-hole gas flow of 20 L/h. The capillary voltage was adjusted to 1300.0 V for positive ions and 1500.0 V for negative ions. The voltage for the sample cone hole was set to 60.0 V for positive ions and 70.0 V for negative ions. Leucine enkephalin online quality correction was performed by the LockSprayTM calibration system. The data collection range was m/z100∼1,500 Da with full scanning.

#### 2.6.2 Metabolomics data analysis

The mass spectrum data were processed by Progenesis QI and EZ info. Orthogonal partial least squares identification analysis (OPLS-DA) was used to predict the reliability and stability of the model for rat stool sample data, and partial least squares identification analysis (PLS-DA) was used to check the differences between groups. Differential ions with variable importance (VIP) > 1 in the projection and *p* < 0.05 in the T-test were selected, and potential biomarkers were identified in combination with the human metabolomics database (HMDB) and Kyoto Encyclopedia of Genes and Genomes database (KEGG), and metabolic pathway enrichment analysis was performed.

### 2.7 16S rDNA high-throughput sequencing

Samples of cecal contents were collected and cryopreserved at −80°C. Bacterial DNA was isolated from cecal contents using the Daisy PowerSoil kit (Qiagen, Hilden, Germany). DNA concentration and integrity were determined by NanoDrop 2000 spectrophotometer (Thermo Fisher Scientific, Waltham, MA, United States) and agar-gel electrophoresis, respectively. To analyze the microbiome composition, a universal primer pair (343F: 5′-TACGGRAGGCAGCAG-3′; 798R: 5′-AGG​GTA​TCT​AAT​CCT-3′) was used to amplify the bacterial DNA. The quality of the amplicon was confirmed through gel electrophoresis. Subsequently, PCR products were purified and quantified using court AMPure XP beads (Beckman Coulter Co., United States) and Qubit dsDNA assay kit, respectively. The concentration of the DNA was adjusted accordingly for sequencing. The original sequencing data were received in FASTQ format and were preprocessed using cutadapt software to identify and remove adapter sequences. After trimming, the paired-end reads were filtered, denoised, and merged with low-quality sequences. The DADA2 (Callahan et al., 2016) algorithm with default parameters in the QIIME2 (Bolyen et al., 2019, 2) software package was used to detect and truncate the reads. As a result, representative sequences and abundance tables for each Amplified Sequence Variant (ASV) were generated. To further analyze the microbial diversity within the cecal content samples, alpha diversity indices such as the Chao1 index (Chao and Bunge, 2002) and Shannon index (Hill et al., 2003) were calculated. These indices provide insights into species richness and evenness in the community. The annotation of all representative reads was performed using the q2-feature-classifier with default parameters and compared against the Silva database Version 138 or Unite for identification of microbial species. Lastly, the amplicon sequencing and analysis of the 16S rRNA gene were conducted by OE Biotech Co., Ltd. (Shanghai, China).

### 2.8 Statistical analysis

The experimental data were analyzed using SPSS19.0 software, and the mean ± SD was used to express the experimental results. The variance in differences among the groups was examined through one-way analysis of variance (ANOVA). A *p*-value of 0.05 or lower was considered indicative of a significant difference between the groups.

## 3 Results

### 3.1 CBD alleviates alveolar inflammation and pulmonary fibrosis in BLM-induced IPF rats

**FIGURE 2:**
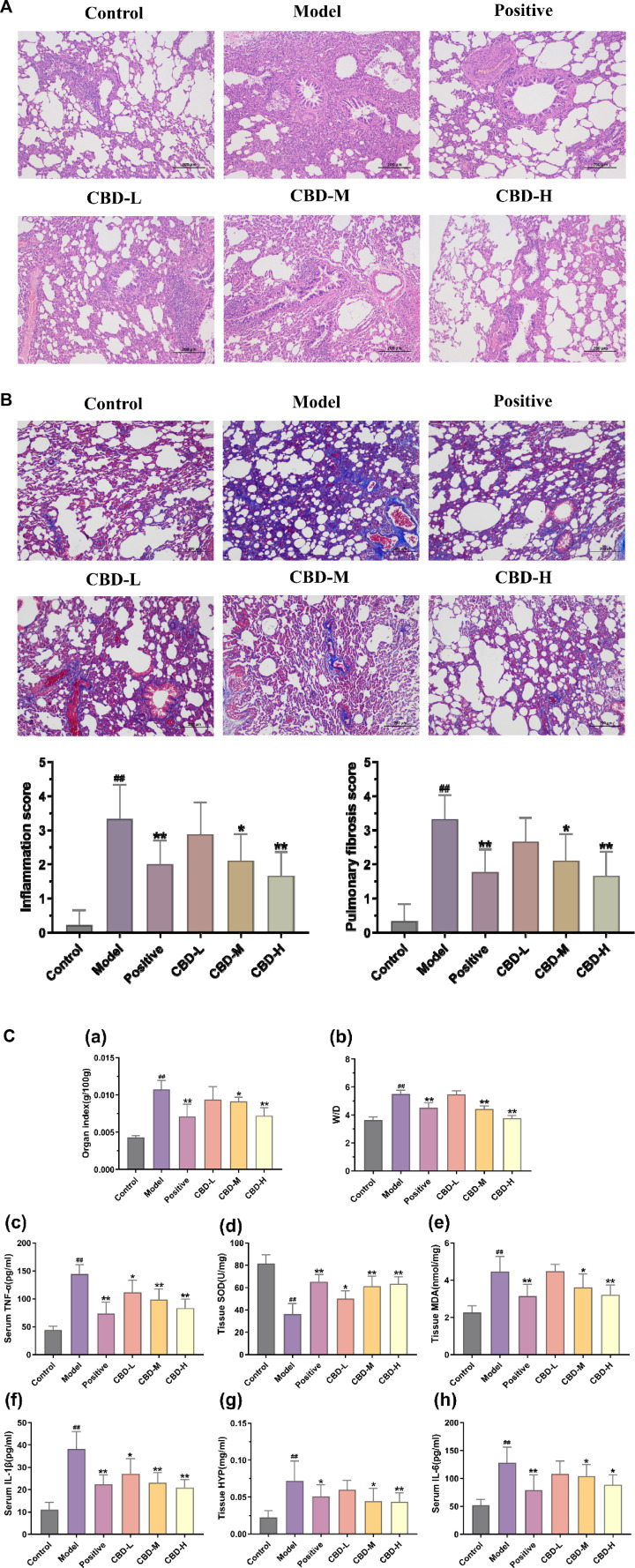
Pathological changes in pulmonary tissue: **(A)** Representative images of H&E staining (×100) and score; **(B)** Representative images of Masson dyeing(×100). **(C)** Analysis of methods for determination of biochemical indexes in each group of rats. (a) Results of the organ index. (b) W/D results. (c) Results of serum TNF-α. (d) Tissue SOD results. (e) Results of the organization of MDA. (f) Results of serum IL-1β. (g) Results of the organization of HYP. (h) Results of serum IL-6. Values represent the mean ± SD. * P < 0.05 and ** P < 0.01 compared to the model group. #P < 0.05 and ##P < 0.01 compared to the sham group.

Compared with the normal control group, 28 days after BLM modeling, the pulmonary tissue of rats showed alveolar structure disorder, abnormal thickening of the alveolar wall, a large amount of matrix deposition, and an increased number of blue collagen fibers and fibroblasts. The CBD and prednisone treatment groups significantly reduced BLM-induced lung injury and fibrosis at day 28, with no significant difference between the CBD and prednisone groups. In addition, both pulmonary fiber and alveolar inflammation scores showed that the degree of pulmonary fibrosis and alveolar inflammation in the CBD treatment group was significantly lower than that in the model group (*p* < 0.05), and the improvement degree in the high-dose group was higher than that in the low-dose and medium-dose groups, indicating that CBD could improve the degree of pulmonary fibrosis and alveolar inflammation in the rats with pulmonary fibrosis (Figures 2A, B).

### 3.2 CBD moderately improves inflammation and oxidative stress in BLM-induced IPF rats

In comparison to the normal control group, the levels of TNF-α, IL-6, and IL-1β in the serum of the model group exhibited a significant increase, showing statistical significance (*p* < 0.01). Conversely, in all CBD groups, the serum contents of TNF-α, IL-6, and IL-1β decreased in contrast to the model group. These differences were statistically significant (*p* < 0.05). Moreover, the reduction of TNF-α and IL-1β in the high-dose CBD group was significantly greater than that in the low and medium-dose groups. This difference was also statistically significant (*p* < 0.01). Additionally, the lung tissue of the model group showed a notable increase in the levels of MDA and HYP, whereas the activity of SOD displayed a significant decrease (*p* < 0.01). In the CBD administration group, the contents of MDA and HYP in lung tissue decreased compared to the model group (*p* < 0.05). Notably, the activities of SOD and MDA in the CBD high-dose group (108 mg/kg) and prednisone acetate group exhibited significant recovery (*p* < 0.01) (Figure 2C).

### 3.3 Metabolomics analysis

#### 3.3.1 Screening of differential metabolites

**FIGURE 3:**
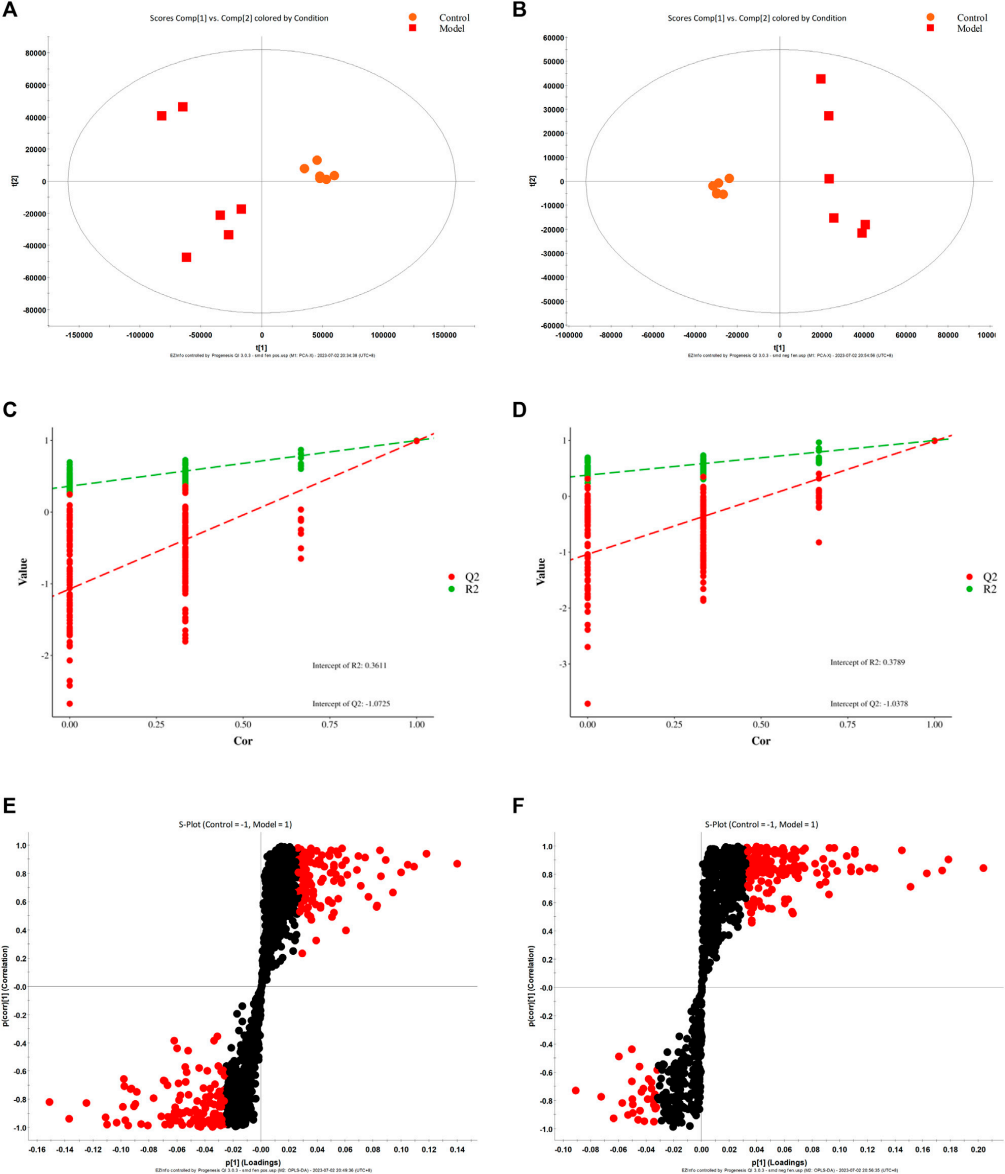
PLS-DA score diagram **(A, B)**, corresponding model verification diagram **(C, D)**, and S-plot score chart **(E, F)** of the control group and model group in positive and negative ion mode.

According to biochemical indexes and histopathological results, high-dose CBD was selected for metabolomics study. Metabolomics studies of rat fecal samples were performed with UPLC-Q-TOF/MS, with one quality control (QC) sample per 10 samples analyzed throughout the injection process to assess stability. The total ion flow chromatogram of QC samples in positive and negative ion modes showed that QC curves overlapped, indicating that the detection system had good stability. Principal component analysis (PCA) and partial least squares discriminant analysis (PLS-DA) were used for multivariate data analysis. The results of the PLS-DA scoring chart are shown in Figures 3A, B. The model group and blank group are well separated, and then the model is verified by permutation test. R2 and Q2 are lower than the rightmost original value from left to right, and Q2 intersects with the Y-axis on the negative semi-axis, indicating that the model does not overfit and has good predictive ability (Figures 3C, D). Based on PLS-DA, S-plots (Figures 3E, F) were further studied, and significant difference metabolites between the normal group and the model group were selected with VIP>1 and *p* < 0.05. A total of 46 different metabolites were identified.

#### 3.3.2 Analysis of fecal metabolic spectrum under CBD intervention

**FIGURE 4:**
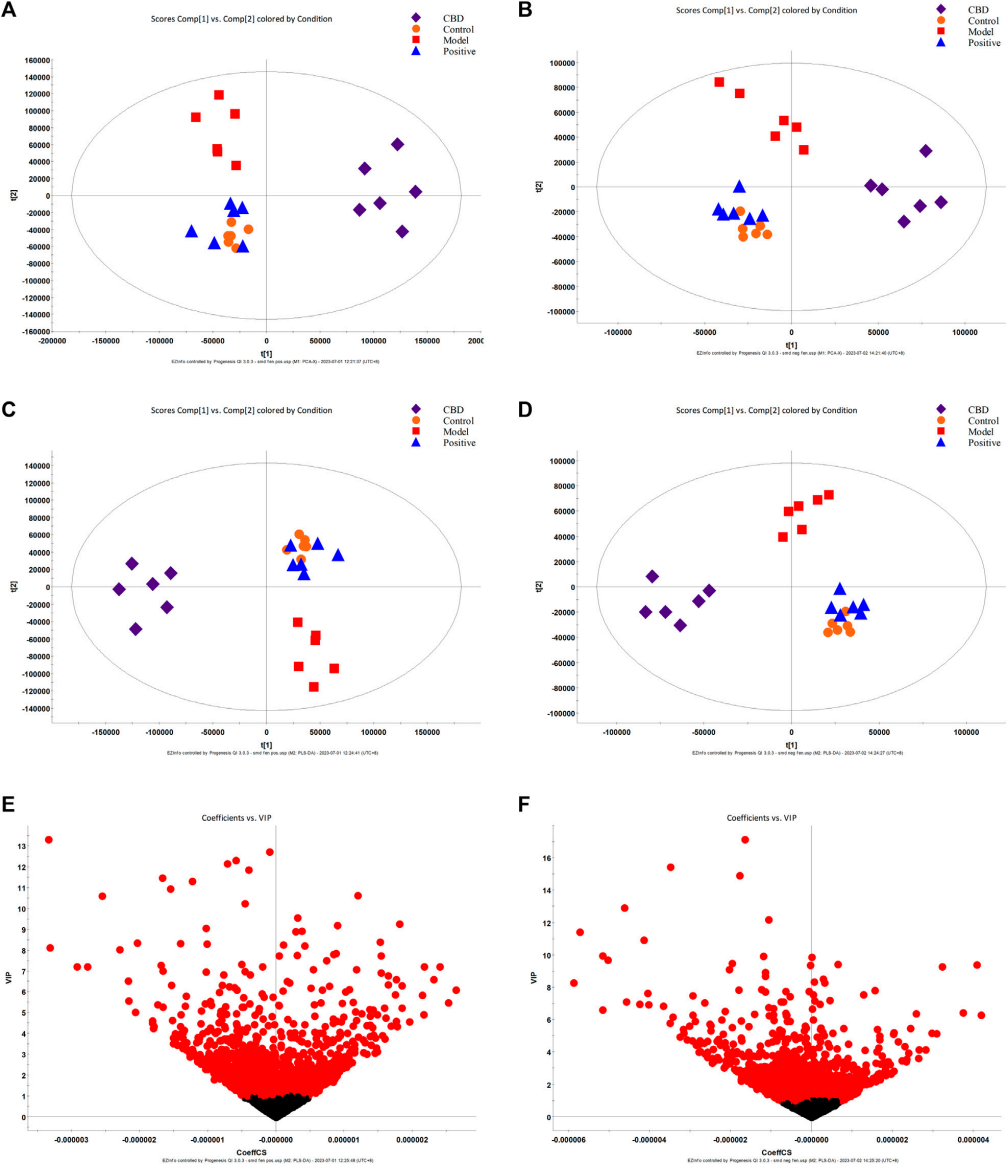
PCA score plot **(A, B)**, PLS-DA score plot **(C, D)**, and S-plot score chart **(E, F)** of the control group, model group, Positive group, and CBD group in positive and negative ion mode.

The results obtained from Principal Component Analysis (PCA) displayed the segregation of the fecal stool metabolic profiles of rats into different groups (Figures 4A, B). Notably, the MON and COD groups exhibited significant separation under both positive and negative ion modes. All identified metabolites were efficiently separated and aggregated. Furthermore, there was a noticeable distinction between the cannabidiol (CBD) group and the MOD group, suggesting that CBD has a positive regulatory effect on the endogenous metabolites of IPF rats. To further investigate the specific effects of each group on the metabolic profile, Partial Least Squares Discriminant Analysis (PLS-DA) was employed to maximize the separation between groups (Figures 4C, D). The obtained results indicated significant differences in metabolite composition between the model group and the CON group. Moreover, the CBD group exhibited an even greater separation from the MOD group and displayed closer proximity to the CON group. By analyzing the Variable Importance in Projection (VIP) scores (Figures 4E, F), it becomes evident that ions closer to the top on both sides of the “V” distribution contribute to a larger extent in driving the trajectory of the metabolic profile, while ions farther away from the top contribute less. The VIP plot enables the selection of markers, denoted by the red color, with most ions being concentrated near the origin. It is these few ions that deviate from the origin that drive the observed differences between groups. Based on the criterion of VIP > 1, potential biomarkers can be effectively screened.

#### 3.3.3 Analysis of relevant metabolic pathways

**FIGURE 5:**
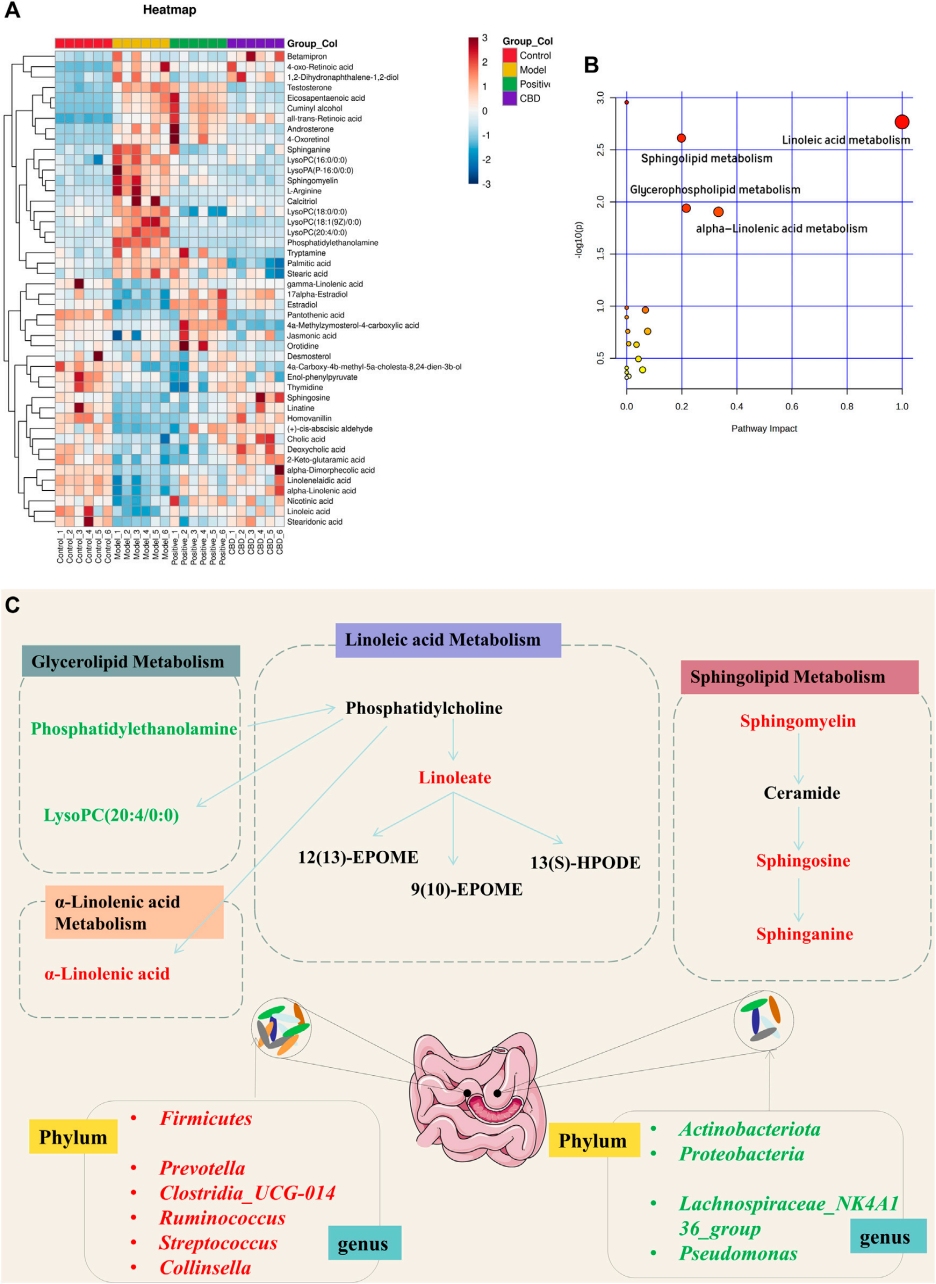
**(A)** Heat map of the differentially abundant metabolites in all groups. The degree of color saturation determines a difference in metabolite expression values between groups. Blue and red indicate the down-regulation and upregulation of MOD and CON expression, respectively. **(B, C)** Metabolic pathway analysis of crucial biomarkers.

To examine the fluctuation of CBD concentration in 46 biomarkers, we conducted a heat map clustering analysis. The analysis revealed the varying patterns of different metabolites in each group, as presented in Figure 5A. A significant alteration in metabolite expression was observed in the model group in comparison to the normal group. However, the CBD group demonstrated the ability to counteract the modified metabolite levels observed in the MOD group. The *p*-value heat map displayed notable variations between the model and blank groups, as well as between the CBD and model groups. Our investigation demonstrated that when compared to the CON group, the expression of 22 distinct metabolites was markedly upregulated, while 24 metabolites displayed significant downregulation in the MOD group. Conversely, the CBD group exhibited downregulation in 22 metabolites and upregulation in 24 metabolites (*p* < 0.05) (Table 1).

**TABLE 1:** Differential metabolites and identification results in feces of control group and model group.

No.	m/z	RT	VIP	Accepted Compound ID	Accepted description	Chemical formula	Trend (M/C)	Trend (CBD/M)
MOD vs. CON—Positive ion mode								
1	524.3721779	10.9483	5.46494	HMDB0010384	LysoPC(18:0/0:0)	C_26_H_54_NO_7_P	↑	↓
2	496.3403241	9.6576	5.23954	HMDB0010382	LysoPC(16:0/0:0)	C_24_H_50_NO_7_P	↑	↓
3	260.1243408	0.41125	1.42272	HMDB0038516	Linatine	C_10_H_17_N_3_O_5_	↓	↑
4	165.0551008	0.64695	3.09474	HMDB0012225	Enol-phenylpyruvate	C_9_H_8_O_3_	↓	↑
5	220.1183701	1.352116667	2.89193	HMDB0000210	Pantothenic acid	C_9_H_17_NO_5_	↓	↓
6	279.2329643	10.10745	6.30105	HMDB0030964	Linolenelaidic acid	C_18_H_30_O_2_	↓	↑
7	279.232538	9.193433333	3.92259	HMDB0001388	alpha-Linolenic acid	C_18_H_30_O_2_	↓	↑
8	281.2482	9.41	2.37703	HMDB0000673	Linoleic acid	C_18_H_32_O_2_	↓	↑
9	124.0396934	0.547016667	1.86816	HMDB0001488	Nicotinic acid	C_6_H_5_NO_2_	↓	↑
10	211.1336099	2.857	1.47174	HMDB0032797	Jasmonic acid	C_12_H_18_O_3_	↓	↑
11	249.149555	3.176633333	1.38857	HMDB0303940	(+)-cis-abscisic aldehyde	C_15_H_20_O_3_	↓	↑
12	273.1854366	5.872183333	1.49869	HMDB0000429	17alpha-Estradiol	C_18_H_24_O_2_	↓	↑
13	273.1854748	6.134733333	1.53055	HMDB0000151	Estradiol	C_18_H_24_O_2_	↓	↑
14	273.221961	6.5137	3.28136	HMDB0000031	Androsterone	C_19_H_30_O_2_	↑	↓
15	301.2168962	6.5065	3.22035	HMDB0012329	4-Oxoretinol	C_20_H_28_O_2_	↑	↓
16	375.2889103	7.318933333	2.19236	HMDB0000626	Deoxycholic acid	C_24_H_40_O_4_	↓	↑
17	443.3534141	9.72185	1.71234	HMDB0001181	4a-Carboxy-4b-methyl-5a-cholesta-8,24-dien-3b-ol	C_29_H_46_O_3_	↓	↑
18	303.2326896	11.41801667	8.11816	HMDB0001999	Eicosapentaenoic acid	C_20_H_30_O_2_	↑	↓
19	239.2377016	12.13921667	1.25757	HMDB0000220	Palmitic acid	C_16_H_32_O_2_	↑	↓
20	267.2689637	13.40715	1.00923	HMDB0000827	Stearic acid	C_18_H_36_O_2_	↑	↓
21	399.3259154	10.1986	2.36038	HMDB0001903	Calcitriol	C_27_H_44_O_3_	↑	↓
22	443.3524799	10.81978333	4.93951	HMDB0006927	4a-Methylzymosterol-4-carboxylic acid	C_29_H_46_O_3_	↓	↓
23	277.2171	9.36	1.59643	HMDB0006547	Stearidonic acid	C_18_H_28_O_2_	↓	↑
24	279.2326771	11.37518333	3.80514	HMDB0004670	alpha-Dimorphecolic acid	C_18_H_32_O_3_	↓	↑
25	261.2220695	10.477	1.09251	HMDB0003073	gamma-Linolenic acid	C_18_H_30_O_2_	↓	↑
26	385.3464928	10.45558333	1.14543	HMDB0002719	Desmosterol	C_27_H_44_O	↓	↑
27	301.216959	10.37706667	1.76909	HMDB0031817	Cuminyl alcohol	C_10_H_14_O	↑	↓
28	271.2064185	10.00038333	1.23567	HMDB0000234	Testosterone	C_19_H_28_O_2_	↑	↓
29	301.2169715	9.102216667	4.26086	HMDB0001852	all-trans-Retinoic acid	C_20_H_28_O_2_	↑	↑
30	300.2905781	8.588216667	2.18291	HMDB0000252	Sphingosine	C_18_H_37_NO_2_	↓	↑
31	431.2771544	6.134733333	1.16411	HMDB0000619	Cholic acid	C_24_H_40_O_5_	↓	↑
32	297.1850747	6.100683333	1.33434	HMDB0006285	4-oxo-Retinoic acid	C_20_H_26_O_3_	↑	↓
33	149.0600879	5.56515	1.59227	HMDB0005175	Homovanillin	C_9_H_10_O_3_	↓	↑
34	180.1022107	3.776316667	2.16326	HMDB0060335	1,2-Dihydronaphthalene-1,2-diol	C_10_H_10_O_2_	↑	↑
35	176.0701606	3.633533333	1.20156	HMDB0034250	Betamipron	C_10_H_11_NO_3_	↑	↑
36	260.1243065	0.561283333	1.06424	HMDB0000273	Thymidine	C_10_H_14_N_2_O_5_	↓	↑
37	300.2906	11.16	3.29904	HMDB0012095	Sphingomyelin	C_47_H_95_N_2_O_6_P	↑	↓
38	302.3064	8.87	1.87497	HMDB0000269	Sphinganine	C_18_H_39_NO_2_	↑	↓
39	566.3469	9.97	1.54872	HMDB0002815	LysoPC[18:1(9Z)/0:0]	C_26_H_52_NO_7_P	↑	↓
MOD vs. CON—Negative ion mode								
1	303.233	1.189733333	1.76543	HMDB0010395	LysoPC(20:4/0:0)	C_28_H_50_NO_7_P	↑	↓
2	283.2643	7.34	2.65433	HMDB0060501	Phosphatidylethanolamine	C_41_H_82_NO_8_P	↑	↓
3	347.217107	6.41315	1.4273	HMDB0000517	L-Arginine	C_6_H_14_N_4_O_2_	↑	↓
4	439.2451664	9.0732	2.27467	HMDB0011154	LysoPA(P-16:0/0:0)	C_19_H_39_O_6_P	↑	↓
5	289.0664494	3.46905	2.85598	HMDB0001552	2-Keto-glutaramic acid	C_5_H_7_NO_4_	↓	↑
6	87.0512667	1.887416667	2.4044	HMDB0000788	Orotidine	C_10_H_12_N_2_O_8_	↓	↓
7	159.0925687	5.615116667	1.44012	HMDB0000303	Tryptamine	C_10_H_12_N_2_	↑	↓

Metabolic pathway Met PA analysis was performed for 46 difference-rich metabolites in the model group and the blank group to identify key biomarkers in the metabolic pathway. As shown in Figure 5B, four metabolic pathways (−log P > 2, impact > 0.02) are mainly involved in linoleic acid metabolism, glycerolipid metabolism, linolenic acid metabolism, and sphingolipid metabolism. These pathways are believed to be closely related to pulmonary fibrosis, and seven key differential metabolites have been identified from these pathways, including Linoleate; Sphingosine; Sphinganine; Sphingomyelin; Phosphatidylethanolamine; LysoPC (20:4/0:0); α-Linolenic acid (Figure 5C).

### 3.4 16S rDNA high-throughput sequencing

#### 3.4.1 Evaluation of sequence diversity

**FIGURE 6:**
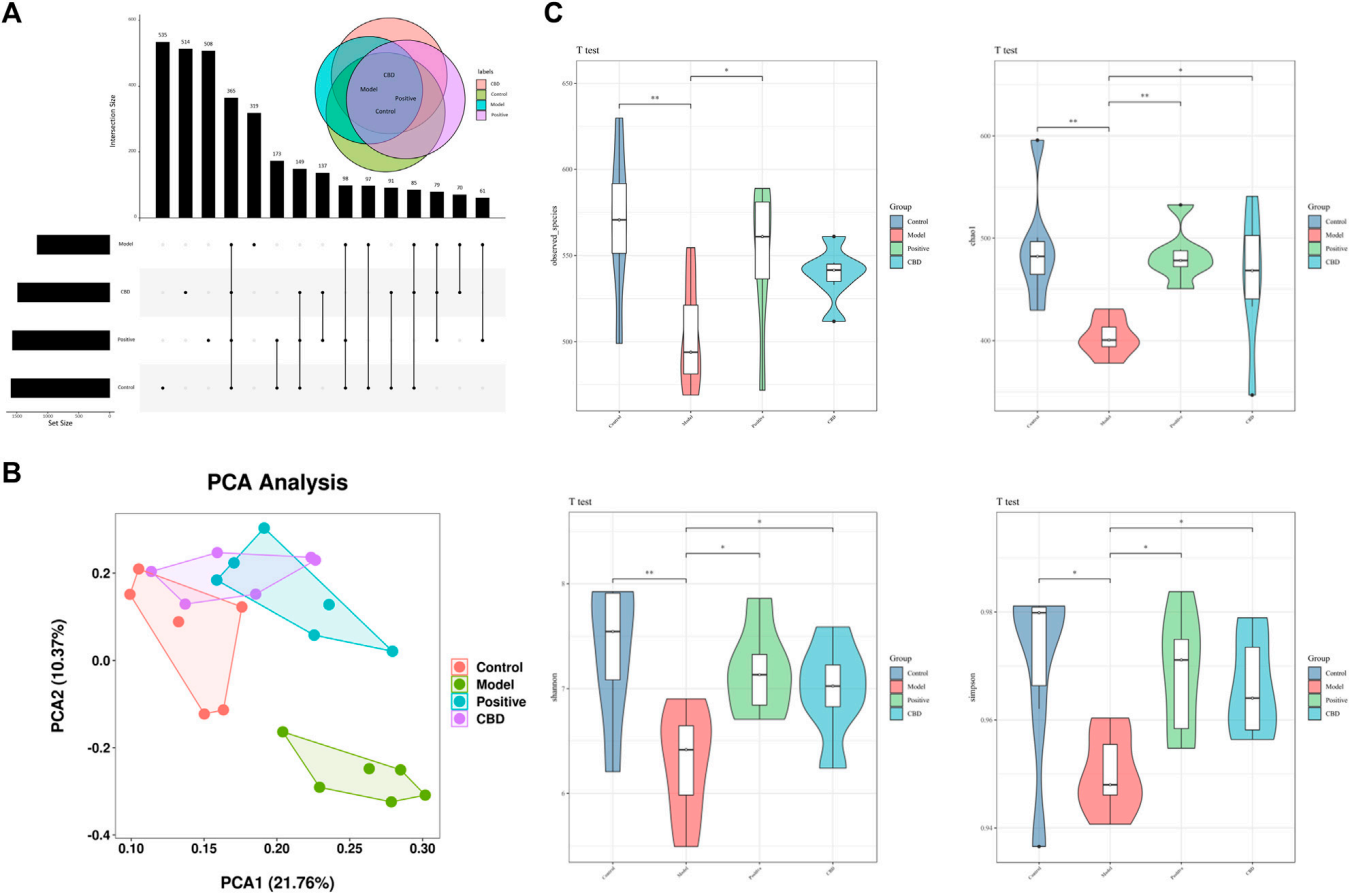
**(A)** Venn diagram depicting the distribution of ASVs among different groups. **(B)** PCA score plot of the control, model, Positive, and CBD groups. **(C)** Intestinal microbial Alpha diversity of Chao1, Simpson, Shannon and Observed species in all groups. Statistical significance was calculated with ANOVA. *p < 0.05 and **p < 0.01 compared to the model control group. #*p* < 0.05 and ##*p* < 0.01 compared to the control group.

As shown in the Venn diagram in Figure 6A, there were 96 ASVs in the model group and the blank group, and the number of ASVs in the blank group was higher, indicating that bleomycin-induced IPF reduced the abundance of intestinal microbiota, while there were 191 ASVs in the CBD group and the blank group, indicating that CBD restored the microbiota of bleomycin-treated rats. Principal Component Analysis (PCA) was employed as the analytical technique to investigate the disparities in the intestinal microbiota among the different experimental groups. As demonstrated in Figure 6B, a marked dissimilarity in microbial composition was observed between the model group and the blank group, suggesting a considerable difference. Additionally, a substantial distinction was found between the CBD group and the model group, with partial overlap between the CBD group and the blank group. These outcomes indicate a greater similarity in microbial composition and structure, accompanied by minimal dissimilarities among the samples. The measurement frequently employed to represent the abundance and variety of species in a given sample is known as the Alpha diversity index. Chao1 and Observed species serve as indices for computing community richness, while Shannon and Simpson serve as indices for computing community diversity. When comparing the model group to the blank group, the abundance and uniformity indices displayed lower values. However, following the administration of CBD, these indices experienced a noticeable increase, especially in the case of Chao1, Shannon, and Simpson (Figure 6C).

#### 3.4.2 Analysis of intestinal microbial composition

**FIGURE 7:**
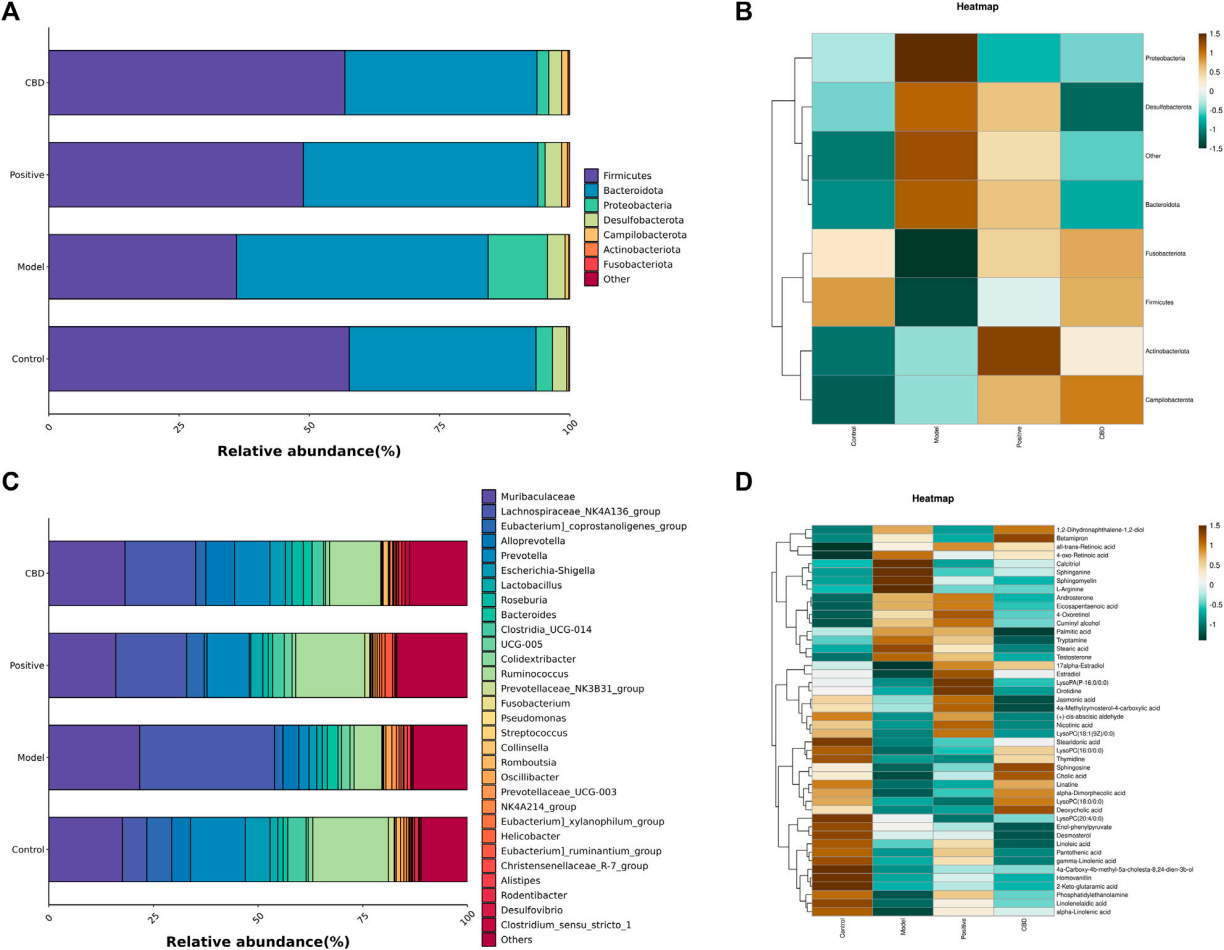
**(A)** Top 7 phyla species composition. **(B)** Heat map of the top7 phyla. **(C)** Top 30 genera species composition; and **(D)** heat map of the top 30 genera. Bright green indicates a lower abundance across species, whereas a bright brown indicates a higher abundance across species.

As shown in Figures 7A, C, The top 8 predominant phyla in relative abundance are *Fusobacteriota, Actinobacteriota, Campilobacterota, Desulfobacterota, Proteobacteria, Bacteroidota, Firmicutes*, The top 30 dominant genera are *Muribaculaceae, Lachnospiraceae_NK4A136_group, [Eubacterium]_coprostanoligenes_group, Prevotella, Alloprevotella, and Clostridia_UCG-014*. Based on the heat maps represented in Figures 7B, D, noteworthy variations were observed in microbial community dissimilarities between the model group and the blank group. Moreover, the administration of CBD and prednisone exhibited partial amelioration of bleomycin-induced dysbiosis.

#### 3.4.3 Difference analysis among groups

**FIGURE 8:**
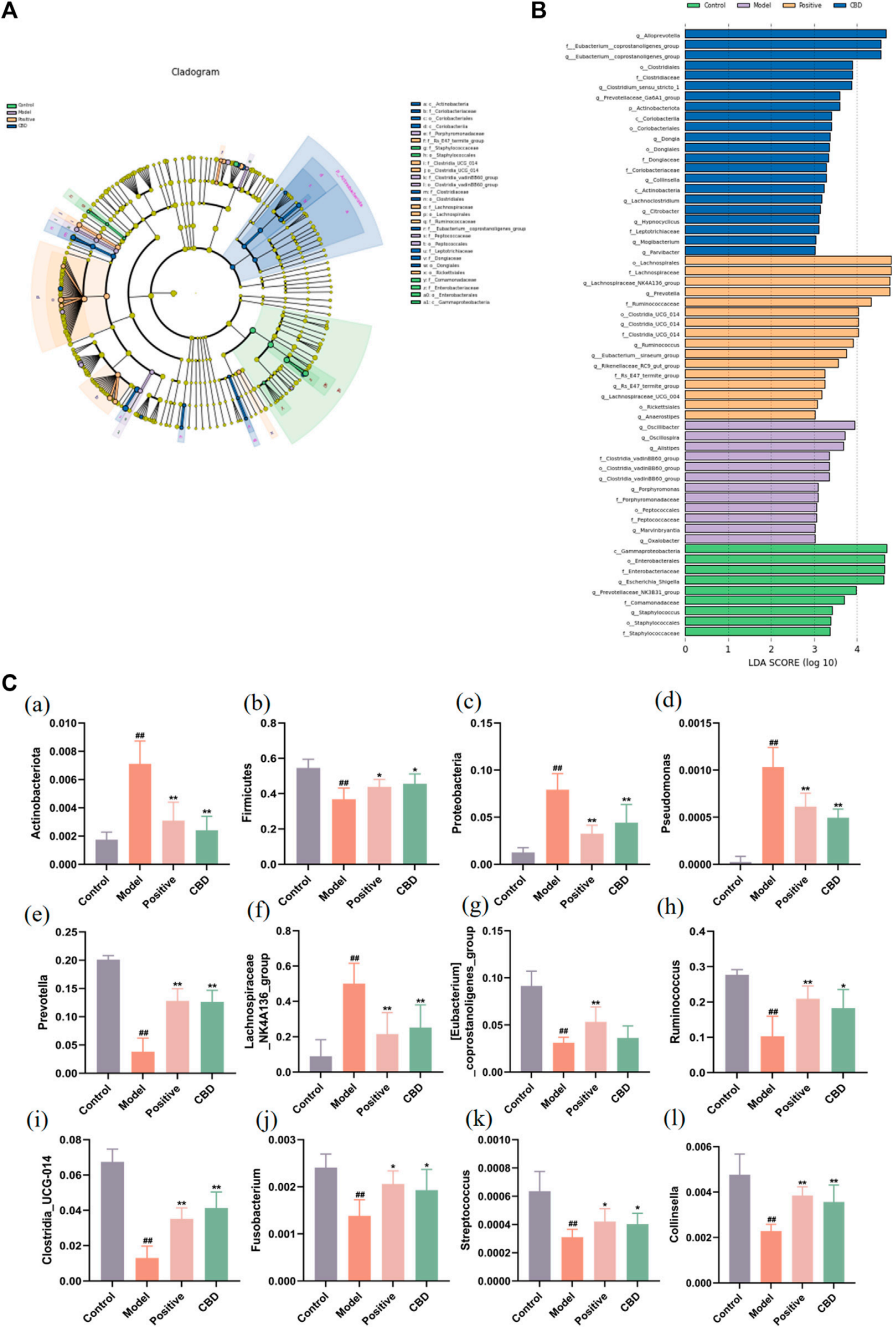
Analysis of the LEfSe. **(A)** Histogram of LDA value distribution. The longer the length, the higher the degree of influence. **(B)** Cladogram. The circles radiating from the inside out represent the classification level from boundary to genus. The diameter of small circles is proportional to the relative abundance. Yellow indicates the biomarkers with no significant difference, and the biomarkers with a significant difference were colored with the group. **(C)** (a–c) Analysis of bacterial flora with a significant difference in phylum level. (d–l) Analysis of bacterial community with a significant difference at the genus level. Values represent the mean ± SD. *p < 0.05 and **p < 0.01 compared to the model group. #*p* < 0.05 and ##*p* < 0.01 compared to the sham group.

To gain better insights into the impact of CBD intervention on the gut microbiota of rats, we conducted a linear discriminant analysis size effect (LEfSe). As shown in Figures 8A, B, the biomarkers were screened with LDA values >3 and *p* < 0.05. The relative abundance of species between groups was used to assess the impact of significantly different species between groups. *Gammaproteobacter, Enterobacterales, Enterobacteriaceae, Escherichia_Shigella, and Prevotellaceae_NK3B31_group* were enriched in the blank group. *Oscillibacter, Oscillospira, Alistipes, Clostridia_vadinBB60_group, Porphyromonadaceae, and Porphyromonas* were enriched in the model group. The prednisone group increased the relative abundance of *Lachnospirales, Lachnospiraceae, Lachnospiraceae_NK4A136_group, Prevotella, and Ruminococcaceae*. In addition, *Alloprevotella, Eubacterium__coprostanoligenes_group, Clostridiales, Clostridiaceae, and Clostridium_sensu_stricto_1* were enriched in the CBD group.

Finally, this study identified the dominant phyla and genera of each group (Figure 8C). Comparatively, at the gate level, the model group exhibited a noteworthy increase in the abundance of *Actinobacteriota, Proteobacteria*, and *Bacteroidota*, whereas the abundance of *Firmicutes* was observed to decline significantly (*p* < 0.01) when compared to the blank group. Following the administration of CBD and prednisone, a marked decrease in the abundance of *Actinobacteriota* and *Proteobacteria* was observed, while the abundance of *Firmicutes* exhibited a significant increase (*p* < 0.05). Furthermore, at the genus level, the MOD group displayed a substantial increase in the abundance of *Lachnospiraceae_NK4A136_group* and *Pseudomonas*. The abundance of *[Eubacterium]_coprostanoligenes_group, Prevotella, Clostridia_UCG-014, Ruminococcus, Fusobacterium, Streptococcus*, and *Collinsella* was significantly decreased (*p* < 0.01). After treatment with CBD and prednisone, the abundance of *Prevotella, Clostridia_UCG-014, Ruminococcus, Fusobacterium, Streptococcus,* and *Collinsella* increased significantly. The abundance of *Lachnospiraceae_NK4A136_group* and *Pseudomonas* decreased significantly. In conclusion, BLM-induced IPF can lead to intestinal microbiota disturbance, while CBD can reverse the disturbance to a certain extent.

### 3.5 Correlation analysis

**FIGURE 9:**
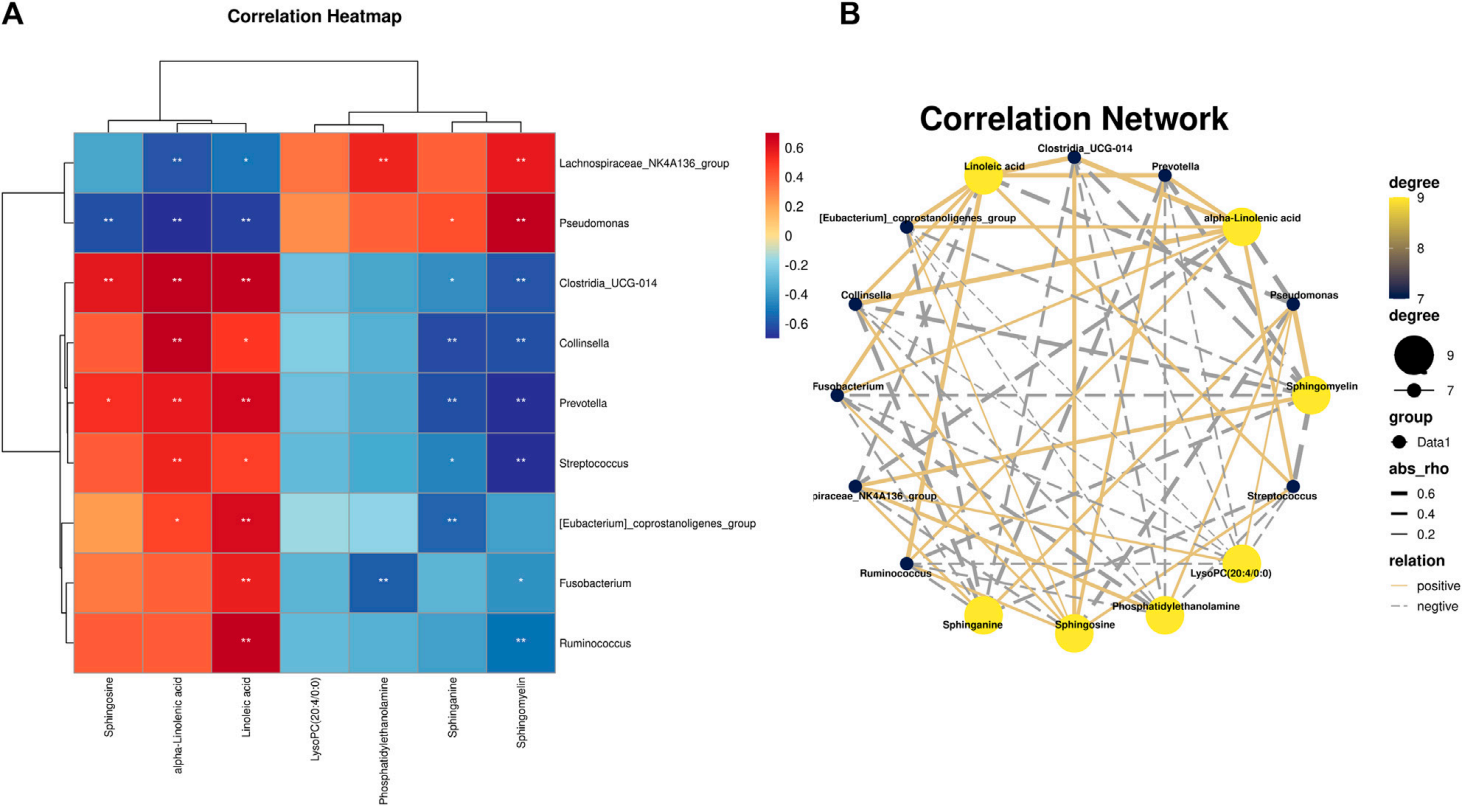
**(A)** Pearson analysis of 7 potential biomarkers and 9 key difference gut microbes. **(B)** Correlation network maps of 7 potential biomarkers and 9 key differentiating gut microbes are shown.

Spearman correlation analysis was used to study the functional relationship between local biomarkers, differential bacteria, and biochemistry. As shown in Figures 9A, B, Metabolites Sphinganine, Sphingomyelin, Phosphatidylethanolamine, and PC(20:4/0:0) were positively correlated with *Lachnospiraceae_NK4A136_group* and *Pseudomonas*, but negatively correlated with *Clostridia_UCG-014, Collinsella, Prevotella, [Eubacterium]_coprostanoligenes_group, Fusobacterium, Ruminococcus, Streptococcus*. In addition, alpha-Linolenic acid, Sphinganine, Linoleic acid was positively correlated with *Clostridia_UCG-014, Collinsella, Prevotella, [Eubacterium]_coprostanoligenes_group, Fusobacterium, Ruminococcus, Streptococcus*, but negatively correlated with *Lachnospiraceae_NK4A136_group* and *Pseudomonas.*

## 4 Discussion

In this study, BLM was used to establish IPF model rats. BLM is a chemotherapy medication that disrupts the normal cell division process, leading to the generation of excessive amounts of unstable molecules known as free radicals. This, in turn, triggers inflammatory responses and causes damage to the lung tissues, including fibroblast activation and subsequent fibrosis. Consequently, BLM is extensively employed in the creation of animal models that mimic IPF (Della Latta et al., 2015; Song et al., 2023). The deposition of extracellular matrix (ECM) is a fundamental pathological characteristic of IPF. Among the various components that make up the ECM, collagen is particularly prominent (Habermann et al., 2020). Furthermore, hydroxyproline, a constituent of collagen, plays a crucial role in its composition (Cao et al., 2022). Through the utilization of H&E and Masson staining techniques, we observed pronounced fibrosis, structural impairment, and the presence of elongated fiber bands in the lungs of rats in the model group. Additionally, biochemical analysis demonstrated a notable elevation in the levels of hydroxyproline. Prednisone, a hormone-based pharmaceutical commonly used for the clinical treatment of IPF (Kooistra et al., 2023), was employed as the reference drug in numerous previous animal studies investigating IPF. Our findings indicate that there was no significant disparity between the high-dose CBD group and the positive control group in terms of their capacity to enhance lung permeability and ameliorate the pathological alterations observed in rats with IPF. This suggests that CBD possesses the therapeutic potential for the treatment of IPF.

Furthermore, IPF (Moss et al., 2022; Savin et al., 2022) is characterized by persistent inflammation, which is a crucial pathological alteration. Fibroblasts undergo constant activation and release substantial quantities of extracellular matrix (ECM) during chronic inflammatory conditions, ultimately resulting in fibrosis and the degradation of regular alveolar structures. Past investigations have indicated the significant involvement of macrophages in IPF (Kishore and Petrek, 2021). In the presence of inflammation, macrophages can swiftly migrate toward the site of inflammation, release numerous inflammatory mediators, and stimulate fibroblast activation and replication (Buechler et al., 2021; Du et al., 2022). ELISA results showed that compared with the control group, there were a large number of pro-inflammatory cytokines in the serum of IPF model rats, which was consistent with the results of other studies (Qin et al., 2021; Chen et al., 2022). The results of the study showed that CBD reduced the inflammatory response in IPF rats.

Oxidative stress is one of the important pathogenesis of IPF (Otoupalova et al., 2020; Forman and Zhang, 2021). This study assessed lung oxidative stress by measuring oxidative stress-related enzyme activities and levels of peroxidation markers. The findings revealed that the model group had a decrease in SOD activity, an enzyme responsible for combating oxidative stress, and an increase in MDA level, a marker of peroxide, suggesting that the lungs experienced severe oxidative stress. However, our study discovered that CBD could improve the antioxidant ability of rats with IPF, thereby reducing damage to lung tissues caused by the peroxidation of lipids.

Metabolomics analysis showed that CBD-H regulates 17 key biomarkers in the IPF model, including Linoleate; Sphingosine; Sphinganine; Sphingomyelin; Phosphatidylethanolamine; LysoPC(20:4/0:0); α-Linolenic acid, etc. These metabolites are related to linoleic acid metabolism, glycerol phospholipid metabolism, linolenic acid metabolism, sphingolipid metabolism, and other pathways, and may inhibit the progression of pulmonary fibrosis. Linoleic acid participates in the formation of phospholipids and maintains the fluidity of the cell membrane. The linoleic acid released by phospholipids in cell membranes can form metabolites involved in cell signaling pathways through the action of various enzymes. Linoleic acid is a precursor to arachidonic acid, which produces prostaglandins and leukotrienes with inflammatory potential (Sonnweber et al., 2018; Wang et al., 2019). The results suggest that CBD may improve pulmonary fibrosis and maintain homeostasis by slowing down linoleic acid metabolism.

Lipids are composed of a variety of molecules that play a key role in cellular energy storage, structure, and signaling (Brohée et al., 2021). Plasma plays an indispensable role in immune response by influencing immune response through the release of lipid-derived mediators (Balboa et al., 2019). In recent years, more and more attention has been paid to the role of lipids in lung and respiratory diseases, including cystic fibrosis, asthma, and chronic obstructive pulmonary disease, all of which are associated with metabolic abnormalities, such as research results demonstrating changes in glycerophospholipid metabolism in IPF patients (Yan et al., 2017). Glycerophospholipids are the main components of biofilms and play a crucial role in the normal function of cells (Powers and Trent, 2019). Glycerol phospholipid metabolism produces a variety of metabolites, such as phosphatidylcholine (PC), phosphatidylethanolamine (PE), lysophosphatidylethanolamine (Lyso PE), lysophosphatidylcholine (Lyso PC), etc. The changes of these metabolites will also affect the normal metabolism (Stephenson et al., 2019). Lyso PC is a precursor of lysophosphatidic acid, which can induce pulmonary fibrosis by mediating vascular leakage and fibroblast migration and proliferation (Corte et al., 2021). Ethanolamine undergoes phosphorylation by ethanolamine kinase, resulting in the formation of phosphoethanolamine (Lykidis et al., 2001). Subsequently, phosphoethanolamine and phosphocholine cytidylyl transferase collaborate to generate high-energy donor CDP-ethanolamine (Fullerton et al., 2007), while 1, 2-diacylglycerol ethanolamine phosphotransferase utilizes the energy supplied by CDP-ethanolamine to bind ethanolamine to the diacylglycerol in the membrane, leading to the formation of PE (Calzada et al., 2016). PE underwent three successive methylation reactions to generate PC, which is called the PE methyltransferase pathway (Ridgway and Vance, 1987). PC can also generate CDP-choline through choline phosphorylation and choline cytidylyl phosphate transferase, and 1, 2-diacylglycerol choline phosphotransferase catalyzed the exchange of cytidine 5'-monophosphate for diacylglycerol formation, a process known as the Kennedy pathway (Sonkar et al., 2019). PC, as the most abundant phospholipid in animals, is involved in several important metabolic pathways, and abnormal levels of PC have been found in many cancer diseases. For example, the hydrolysis of PC produces lipid mediators. The production of these lipid mediators is beneficial to the survival, proliferation, and growth of cancer cells. Immunomodulatory cell-to-cell crosstalk ultimately leads to resistance to therapy (Saito et al., 2022). The alveoli, the most active lipid metabolizer in the lung, requires surfactant lipids for the synthesis of respiration (Agudelo et al., 2020), and PC and PE are the key lipids of pulmonary surfactants, and the total saturation of silicosis rat lungs increases (Srivastava and Misra, 1986). Xia et al. (2021) also confirmed that the contents of PC and PE in bleomycin-induced pulmonary fibrosis rats were increased. This is consistent with the results of this study. After CBD treatment, LysoPC(18:0/0:0), LysoPC(16:0/0:0), PE, Lyso PC(20∶4/0∶0), LysoPC[18:1(9Z)/0:0] were significantly decreased compared with the model group. Therefore, it is speculated that CBD may reduce the synthesis of PC by slowing down the PE methyltransferase pathway and the Kennedy pathway in glycerophospholipid metabolism, and regulate inflammation and endothelial cells. Proliferation and apoptosis, thus affecting the development of IPF. α-linolenic acid has been reported to have protective effects on cardiovascular nerves, anticancer, anti-osteoporosis, anti-inflammatory and antioxidant effects (Yuan et al., 2022). Studies have found that flaxseed oil can effectively protect rat lung tissue from bleomycin-induced pulmonary toxicity, promote increased lumen patency and decreased pulmonary septal thickness, reduced inflammatory cell infiltration, delayed edema formation, reduced vascular inflammation and pulmonary and peribronchial fibrosis (Lawrenz et al., 2012).

Extensive research has been conducted on the potential involvement of the gut microbiota in IPF, which has demonstrated a significant correlation with inflammation (Bhattacharya et al., 2022). This study employed 16S rRNA sequencing analysis to ascertain the impact of CBD on the structure and composition of intestinal microbiota in IPF rats.

This article further elucidates the changes in the intestinal microbiome of the IPF model after CBD-H intervention to identify the microbiome associated with IPF progression, and the top few bacteria with the highest relative total abundance at the genus level were selected for comparison. The IPF rats exhibited a significant decrease in the relative abundance of *[Eubacterium]_coprostanoligenes_group, Prevotella, Clostridia_UCG-014, Ruminococcus, Fusobacterium, Streptococcus* and *Collinsella* while witnessing a notable increase in the relative abundance of *Lachnospiraceae_NK4A136_group* and *Pseudomonas*. CBD may significantly increase *Prevotella, Clostridia_UCG-014, Ruminococcus, Fusobacterium, Streptococcus, and Collinsella* and decrease the abundance of *Lachnospiraceae_NK4A136_group* and *Pseudomonas. Prevotella* is often considered a bacterium associated with a healthy plant-based diet, acting as a “probiotic” in the human body. The decline of *Prevotella* has been associated with certain diseases. *Ruminococcus* produces metabolites in the form of glucomannan polysaccharide. Ruminalcoccal polysaccharides can activate immune system cells, including tumor necrosis factor (TNF-α), while ruminal *bacillus* plays a crucial role in the synthesis of single-chain fatty acids (SCFAs), which serve as the primary energy source for colon cells. Patients with end-stage renal disease experience a substantial decrease in the production of SCFAs by bacteria (Zhu et al., 2020). Yu et al. (2021) discovered a positive association between Rumenococcaceae and liver health status, while a negative association was observed with factors related to injuries. Furthermore, Ruminococcaceae may impede the progression of chronic kidney disease (Hsiao et al., 2021). Consistent with the results of this article, *Ruminococcus* can be used for further research as a pathogenic bacterium. However, there is debate about the effects of *Lachnospiraceae* on the respiratory system. Some studies have reported the protective effect of *Lachnospiraceae* in the respiratory system to a certain extent. Zhao et al. (2021) found in the study of particulate matter-induced lung injury in mice that the *Lachnospiraceae_NK4A136_group* may be the core intestinal microbe playing a protective role. However, other studies have noted that there is no negative association between *Lachnospiraceae* and respiratory diseases. Li et al. (2019) found that an increased abundance of *Lachnospiraceae_NK4A136_group* was associated with high levels of IL-17, which is known to promote inflammation by activating CD4 ^+^ T cells. Furthermore, there was a significant positive correlation between the *Lachnospiraceae_NK4A136_group* and IgE and IL-33 (Wang et al., 2022). The results of this experiment showed that the relative abundance of Lachnospiraceae_NK4A136_group in the intestinal microbiota of IPF mice was significantly increased and reversed by CBD intervention. This appears to be at odds with prior findings, and the discrepancies between the studies may be due to disparities in environment, geography, and dietary practices. Furthermore, varying sampling techniques may also be a contributing factor to the disparate outcomes. Furthermore, it is linked to the diversity of each individual’s pulmonary microbiome. Hence, it is imperative to delve deeper into the precise function of the *Lachnospiraceae_NK4A136_group* in the context of lung disease in subsequent research. In addition, CBD increases the abundance of beneficial bacteria, such as *Fusobacterium, Streptococcus, and Collinsella.* In summary, CBD can effectively inhibit inflammatory cell infiltration and pulmonary fibrosis by regulating intestinal microbiota.

What is the mechanism by which CBD affects intestinal microbes and alters the metabolic composition of the host stool? It is widely acknowledged that small molecule metabolites play a crucial role in linking the gut-lung axis. This article conducted a correlation analysis between intestinal microbiota and potential metabolic markers and found that there is a significant correlation between the two. CBD can control the levels of *Lachnospiraceae_NK4A136_group, Pseudomonas, Clostridia_UCG-014, Collinsella, Prevotella, [Eubacterium]_coprostanoligenes_group, Fusobacterium, Ruminococcus,* and *Streptococcus*, thereby reinstating the activity of gut bacteria and reversing the pattern of fecal metabolism. Simultaneously, it has the ability to activate Sphinganine, Sphingomyelin, alpha-Linolenic acid, Sphinganine, Linoleic acid, Diminish Phosphatidylethanolamine and PC (20:4/0:0), control the metabolism of linoleic acid, glycerol phospholipid, linolenic acid and sphingolipid, shield lung tissue from the consequences of inflammatory elements and ultimately act as an anti-IPF agent.

## 5 Conclusion

In conclusion, this study suggests that CBD treatment can improve pulmonary fibrosis by inhibiting inflammatory responses and oxidative stress. This research employed a combination of pharmacodynamics, 16SrRNA, and metabolomics to investigate the impact of CBD on pulmonary fibrosis, which provided a basis for further exploration into the pathology of IPF and the creation of new pharmaceuticals. Future research could employ targeted metabolomics to ascertain the metabolic control mechanisms of CBD on pulmonary fibrosis. Furthermore, due to the strong correlation between metabolic alterations and the development of pulmonary fibrosis, such as inflammation and oxidative stress, further research can be done with *in vitro* models to gain a better understanding of how CBD hinders inflammation and oxidative stress by influencing metabolism.

## Data Availability

The data presented in the study are deposited in the NCBI repository, accession number PRJNA1036561.
